# Polypoid arteriovenous malformation of the ureter mimicking a fibroepithelial polyp, a case report

**DOI:** 10.1186/s12894-017-0237-z

**Published:** 2017-07-10

**Authors:** C.S. ten Donkelaar, A.C. Houwert, F.J.W. ten Kate, M.T.W.T. Lock

**Affiliations:** University Medical Center Utrecht, Heidelberglaan 100, 3584 CX Utrecht, The Netherlands

**Keywords:** Arteriovenous malformation, Polyp, Ureter, Case report

## Abstract

**Background:**

Arteriovenous malformations (AVM) of the urinary tract are extremely rare. To the best of our knowledge, only three case of AVM of the ureter have been described in the literature so far.

**Case presentation:**

We present an additional, fourth case of an AVM of the ureter, clinically presented as asymptomatic haematuria and an obstructive process in the left ureter. Ureteroscopic evaluation revealed a fibroepithelial polypoid-like lesion in the proximal ureter. After biopsy showed a benign lesion, the lesion was treated with the 2-μm continuous wave (cw) thulium laser. Histopathological examination revealed a polypoid laesion caused by a circumscribed arteriovenous malformation. Almost four years after operation the patient remains asymptomatic and free of recurrence.

**Conclusion:**

Arteriovenous malformations of the urinary tract are extremely rare. We presented a fourth case of a arteriovenous malformation of the ureter.

**Electronic supplementary material:**

The online version of this article (doi:10.1186/s12894-017-0237-z) contains supplementary material, which is available to authorized users.

## Background

Ureteral tumours are rare, and 20% of these tumours are benign [1]. Fibroepithelial polyps (FEP) are the most common benign ureteral tumours. Approximately 220 cases have been described [2, 3]. In recent years, most cases of FEP have been presented in China and Japan, which suggests that this disease is more prevalent in East Asia [4]. Another benign ureteral tumour is the arteriovenous malformation (AVM). AVMs of the urinary tract are also rare: to our knowledge only three cases have been described in the literature, two of which consisted of a polypoid mass [5–7]. We present the fourth case of an AVM of the ureter in a patient with macroscopic haematuria, which mimics a fibroepithelial polyp.

## Case Presentation

**Fig. 1:**
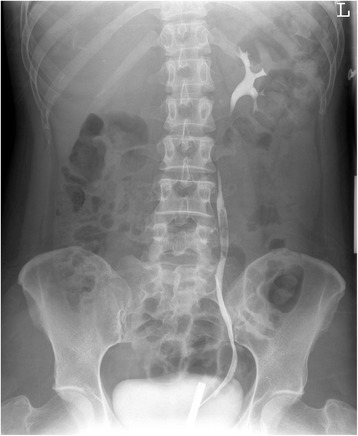
Retrograde pyelography with a filling defect in left ureter from L4-L5

A 41 year old woman was referred to our hospital for asymptomatic macroscopic haematuria, which was discovered during a procedure of in vitro fertilisation. She experienced some discomfort in the middle lower quadrant of the abdomen. There was no history of urinary tract infections or stones. Physical examination findings were within normal limits. Urinalysis showed haematuria, and cytology revealed atypical transitional cells. Ultrasonography of the kidneys presented neither hydronephrosis nor urolithiasis. Abdominal-pelvic computed tomography revealed a thickened ureteral wall of the proximal part of the left ureter. Moreover, a presumed blood clot was observed in the ureter for which there was no suspicion of tumour. Retrograde pyelography revealed a filling defect of the left ureter from L4–L5 [Fig. 1].

**Fig. 2:**
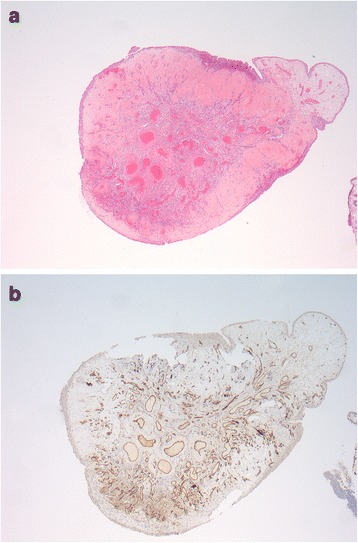
**a**, **b** Hematoxyline-eosine staining and CD31 of the ureteral lesion matching a arteriovenous malformation


Ureteroscopy showed a 3 cm, firm polypoid-like lesion with a glistening appearance. A biopsy of this polypoid-like lesion demonstrated fibrosis, with no signs of malignancy. It was decided to perform ureteroscopic polypectomy to remove the lesion by the 2-μm cw Thulium laser; during the intervention, no bleeding occurred [see Additional files 1 and 2]. A 6–Fr double J-stent was left in place. Histopathological examination revealed a polypoid tissue specimen composed of mucosa without adjacent muscularis propria. The mucosa was covered by uneventful transitional cell epithelium and focally subepithelial hyalinised stroma. The tunica propria presented a circumscribed collection of smaller and larger arterial and venous type blood vessels. Some vessels showed asymmetrical thickening of the muscle wall. These findings were consistent with the diagnosis of AVM [Fig. 2a and b]. The patient had an uneventful recovery. The double J-stent was removed after two weeks, no recurrence was observed in the follow-up with abdominal-pelvic computed tomography and urine cytology during a period of eight years.

Additional file 1: ‘Intro en ureter tumorpeg 2: It is videoclip used for Dutch education where our case is used as illustration for different ureter abnormalities and their treatment. (MPEG 37356 kb)


## Discussion

AVMs of the ureter are very rare. To our knowledge only three cases have been described [5–7]. The first case was a 34 year old woman with intermittent gross haematuria together with mild left flank pain. Urine cytology was initially reported as demonstrating malignant cells; further examination revealed that these were benign cells of non-transitional origin. A retrograde pyelogram showed a filling defect in the mid-ureter. Ureteroscopy revealed a polyp. An excisional biopsy was performed and histological examination demonstrated the presence of an AVM [7]. In the second case, a 19 year old woman presented with repeated total haematuria with intermittent right loin discomfort associated with the attacks of haematuria. Right retrograde pyelography showed a filling defect of the distal end of the ureter. Ureteroscopy revealed an inflamed, ulcerated, ‘cobblestone’-like mucosa and histology confirmed chronic inflammation without evidence of malignancy. Eventually, right renal artery cannulation showed tortuous vessels around the right ureter consistent with AVM, and selective embolization was carried out [6]. The third case was a 29 year old non-Caucasian woman with intermittent gross haematuria and right lower quadrant pain. Urine cytology revealed no malignant cells. Retrograde pyelography showed a filling defect. Uretroscopy was performed and frozen section biopsy revealed benign urothelial mucosa. The polyp was removed using a Greenwald electrosurgical probe [5]. On histopathological examination cases one and three revealed numerous large and small blood vessels superficially located with irregular and asymmetrical walls, confirming the diagnosis of AVM.

Histological examination of the lesion is warranted because on visual inspection it is difficult to distinguish a benign lesion of the ureter from a papillary urothelial neoplasm. Moreover, pre-operatively it is important to know what kind of lesion will be treated so that the most preferable approach can be used. For AVM, laser excision is the best procedure since it diminishes the risk of haemorrhage. Selective embolization is also an option, depending on the location of the AVM lesion. However, excisional biopsy with laser coagulation is indicated when a lesion of unknown origin is treated. In our case (case four), cytology of the urine showed moderate to severe atypia. However, on visual inspection and biopsy a benign lesion was suspected; therefore, in this case laser surgery was the preferred approach.

## Conclusion

Arteriovenous malformations (AVM) of the urinary tract are extremely rare. We presented a fourth case of a arteriovenous malformation of the ureter.

## Additional files

Additional file 2: Epigenetic influences: Environmental and Lifestyle Influences on Genomics, Proteomics and Metabolomics. (PNG 90 kb)

## Abbreviations

AVM: Arteriovenous malformation
cw: Continuous Wave
FEP: Fibroepithelial polyp
