# Supplementary figures and images for: Polypoid arteriovenous malformation of the ureter mimicking a fibroepithelial polyp, a case report
Source: BMC Urol. 2017 Jul 10;17:55. doi: 10.1186/s12894-017-0237-z (PMC5504856; doi:10.1186/s12894-017-0237-z)

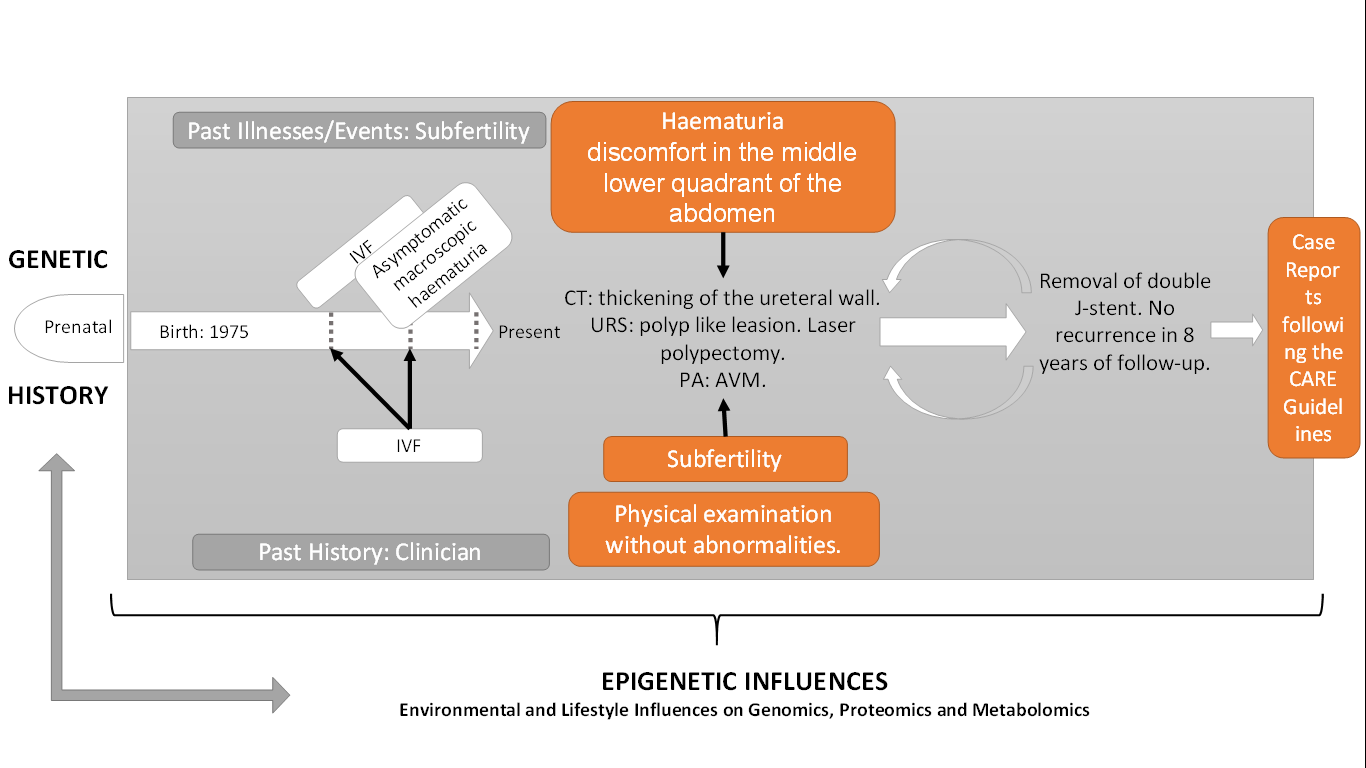

Supplement: Supplementary file 2 — Epigenetic influences: Environmental and Lifestyle Influences on Genomics, Proteomics and Metabolomics. (PNG 90 kb) [file 12894_2017_237_MOESM2_ESM.png]
